# The association between plasma levels of Sestrin2 and risk factors of cardiovascular diseases in healthy and diabetic adults: A study of Qatar Biobank data

**DOI:** 10.17305/bb.2024.11418

**Published:** 2024-11-29

**Authors:** Shahenda Salah Abdelsalam, Muhammad Ammar Zahid, Hicham Raïq, Hanan H Abunada, Ahad E Elsayed, Aijaz Parray, Abdelali Agouni

**Affiliations:** 1 Department of Pharmaceutical Sciences, College of Pharmacy, QU Health, Qatar University, Doha, Qatar; 2 Department of Social Sciences, College of Arts and Sciences, Qatar University, Doha, Qatar; 3 Office of Vice President for Medical & Health Sciences, QU Health, Qatar University, Doha, Qatar; 4 The Neuroscience Institute, Academic Health System, Hamad Medical Corporation, Doha, Qatar

**Keywords:** Sestrin2, SESN2, cardiovascular disease, CVD, cardiometabolic risk, type 2 diabetes mellitus, T2DM

## Abstract

This study examines the association between serum Sestrin2 (SESN2) levels and cardiovascular disease (CVD) risk factors in healthy and diabetic adults, using data from the Qatar Biobank (QBB). A total of 844 participants were included, with 518 in the diabetic cohort and 326 in the healthy cohort. Clinical characteristics, cardiometabolic markers, and SESN2 levels were measured, and binomial logistic regression analyses were conducted to assess the associations between SESN2 and various health indices. Diabetic patients had significantly lower SESN2 levels compared to healthy controls (5.49 ± 5.94 vs 8.25 ± 7.57 ng/mL, *P* < 0.001). A significant negative correlation was observed between SESN2 and HbA1c (−0.19, *P* ═ 0.0006), insulin (−0.19, *P* ═ 0.0006), HOMA-IR (−0.17, *P* ═ 0.0024), C-peptide (−0.18, *P* ═ 0.0012), triglycerides (TG)/HDL ratio (−0.12, *P* ═ 0.0283), and the pulsatility index (PI) (−0.15, *P* ═ 0.006). In healthy individuals, higher SESN2 levels were associated with lower odds of elevated HbA1c (adjusted odds ratio [AOR] ═ 0.33, *P* ═ 0.00), insulin (AOR ═ 0.23, *P* ═ 0.00), HOMA-IR (AOR ═ 0.58, *P* ═ 0.06), C-peptide (AOR ═ 0.56, *P* ═ 0.04), and TG (AOR ═ 0.37, *P* ═ 0.03). In contrast, diabetic patients showed a positive correlation between SESN2 and insulin (0.15, *P* ═ 0.0005), HOMA-IR (0.11, *P* ═ 0.0106), and C-peptide (0.12, *P* ═ 0.0048). Participants in the highest SESN2 tertile had increased risks for high BMI (AOR ═ 1.96, *P* ═ 0.05), high TG (AOR ═ 1.57, *P* ═ 0.04), high NT-proBNP (AOR ═ 7.27, *P* ═ 0.01), and high fibrinogen (AOR ═ 1.92, *P* ═ 0.03). These findings suggest that while high SESN2 levels are cardioprotective in healthy individuals, they may indicate higher cellular stress in diabetics. Determining optimal SESN2 levels could help assess CVD risk, particularly in diabetic patients.

## Introduction

Diabetes has emerged as a significant contributor to the global burden of disease and mortality. In 2017, there were 425 million documented cases of diabetes, a figure projected to rise to 629 million by 2040, with 22 million new cases reported annually [1]. This widespread prevalence can be partly attributed to factors such as unhealthy lifestyle patterns, an aging population, and rising obesity rates in both adults and children. Diabetes-related complications—including coronary heart disease (CHD), stroke, peripheral artery disease (PAD), heart failure (HF), diabetic retinopathy (DR), renal disease, and cardiac autonomic neuropathy (CAN)—significantly amplify the disease’s impact on health. Importantly, individuals with type 2 diabetes mellitus (T2DM) experience cardiovascular disease (CVD) approximately 15 years earlier than their non-diabetic counterparts, making CVD the leading cause of morbidity and mortality within this population [2]. According to a 2023 report by the American Heart Association (AHA), CVD remains a leading global cause of death [3]. Between 2010 and 2020, the number of deaths attributed to CVD rose by 18.71%, reaching approximately 19.05 million. During the same period, the global prevalence of CVD cases increased by 29.01% [3]. CVD is not only a leading cause of mortality but also significantly reduces quality of life. Clinical trials have shown that chronic inflammatory disorders are intricately linked to CVD. Advances in research have identified various inflammatory cells as contributors to vascular oxidative stress, an interaction that likely explains the strong association between atherosclerosis, CVD, and inflammation [4]. Furthermore, the connection between CVD, predominantly atherosclerotic conditions, and oxidative stress has been well established, driven by an overproduction of reactive oxygen species (ROS) [5].

Sestrin2 (SESN2), initially named hypoxia-induced gene 95 (HI95), was identified during efforts to understand the genes that regulate cellular survival and fate under extended periods of hypoxia. SESN2 belongs to a class of highly conserved proteins that are activated by various stressors, including oxidative stress, genotoxic stress, endoplasmic reticulum (ER) stress, hypoxia, and metabolic and energetic stress [6]. It primarily exerts its multifaceted effects by activating key metabolic pathways. Research has shown that SESN2 plays a protective role against oxidative damage in CVDs by regulating ROS levels. By stabilizing nuclear factor erythroid 2-related factor 2 (NRF2)—a transcription factor that activates antioxidant genes—SESN2 enhances the cellular antioxidant defense system. This protective mechanism supports cell survival in conditions such as ischemia/reperfusion injury and HF, while preventing cellular damage associated with metabolic disorders like obesity and insulin resistance [7, 8].

SESN2 also acts as an energy and nutrient sensor. It activates AMP-activated protein kinase (AMPK) during energy scarcity and inhibits mTORC1 to reduce protein synthesis, enabling cells to adapt to energy deficits and nutrient shortages [9]. Furthermore, SESN2 helps maintain cellular homeostasis by promoting autophagy and mitophagy—processes that degrade damaged organelles and selectively remove dysfunctional mitochondria, respectively [10]. These functions are critical for preventing cellular oxidative damage.

In the context of CVD, inflammation plays a key role in disease progression. SESN2 has been shown to prevent the activation of proinflammatory pathways and the formation of foam cells, particularly in diabetic conditions [11, 12]. These findings position SESN2 as a potential therapeutic target for metabolic disorders, including T2DM, obesity, and CVD.

Preliminary studies suggest that SESN2 plays a significant role in the development of chronic diseases, such as diabetes, atherosclerosis, cancer, and other conditions [13]. Examining circulating SESN2 levels may provide valuable insights into the risk of developing CVD, both in healthy individuals and those with T2DM. This could open new avenues for preventing diabetes-induced CVD.

There is an urgent need to further clarify the intricate relationship between metabolic disturbances and CVD, particularly among high-risk groups such as diabetic individuals. The objective of this study is to investigate the role of SESN2 in cardiovascular health among both healthy and diabetic individuals and to evaluate its potential as a biomarker for CVD risk. Specifically, the study compares plasma levels of SESN2 in individuals with and without diabetes and examines the association between CVD risk factors and circulating SESN2 levels in both groups.

## Materials and methods

### Study population

This retrospective observational study investigates the association between SESN2 levels and key cardiovascular risk markers in the Qatari population. Participants included Qatari nationals and long-term residents (≥15 years) recruited from the Qatar Biobank (QBB), a national biorepository that collects biospecimens, clinical and biochemical data, and health and lifestyle information from a large segment of the Qatari population, with longitudinal follow-up for up to five years. Eligible participants were healthy and diabetic adults (aged ≥18) with comprehensive data on metabolic and CVD risk markers, along with plasma samples available for SESN2 level analysis.

The exclusion criteria consisted of the presence of other chronic diseases (e.g., cancer, autoimmune disorders, and genetic conditions), the use of vitamin or mineral supplements, smoking, pregnancy or breastfeeding, and the presence of implantable electrical devices, such as pacemakers, defibrillators, or nerve stimulators. A total of 844 eligible adult individuals were included in the study.

The research investigation examined a comprehensive range of CVD risk markers, including body mass index (BMI), fasting serum glucose, insulin, hemoglobin A1c (HbA1c), C-peptide, HOMA-IR, lipid profile [triglycerides (TG), total cholesterol, low-density lipoprotein cholesterol (LDL), and high-density lipoprotein cholesterol (HDL)], TG-to-HDL ratio (TG/HDL), TG-glucose index (TyG), systolic blood pressure (SBP), diastolic blood pressure (DBP), pulse rate (PR), pulse pressure index (PPI), pulse wave velocity (PWV), and N-terminal (NT)-prohormone B-type natriuretic peptide (NT-proBNP). Furthermore, coagulation parameters, such as prothrombin time (PT), activated partial thromboplastin time (aPTT), international normalized ratio (INR), and fibrinogen were evaluated.

The study also assessed the structure and function of the left and right common carotid arteries using three-dimensional carotid Doppler ultrasonography. This technique analyzed markers indicating blood flow resistance and arterial stiffness or constriction. Key criteria included the pulsatility index (PI) and intima-media thickness (IMT). Elevated PI values may reflect increased arterial stiffness or constriction, while heightened IMT may signal greater susceptibility to CVD. These markers provide valuable insights into carotid artery disease and help identify individuals at an elevated risk of cardiovascular events.

Participants’ PPI and PWV were measured using a Viacorder (Viacor CardioMPO), a device designed to record arterial pulse waveforms at two distinct body locations. PPI was computed by multiplying heart rate by stroke volume, while PWV quantified the speed of arterial pulse wave propagation between the measurement points. Both metrics are crucial for evaluating arterial stiffness and identifying individuals at higher risk for developing CVD.

### Anthropometric and biochemical measurements

Data collection was conducted at the QBB clinic by qualified and well-trained technicians and nurses. Height and weight were measured using a calibrated scale and a wall-mounted stadiometer (Seca, Hamburg, Germany). Participants were instructed to wear light clothing and remove their shoes during the measurements. SBP and DBP readings were taken three times using a mercury sphygmomanometer, and the average values were recorded. PR was determined using a digital device.

After an overnight fast, blood samples were collected from each participant and analyzed at the clinical chemistry laboratories of Hamad Medical Corporation (HMC). Standard automated laboratory protocols were followed to measure fasting glucose, HbA1c, insulin, total cholesterol, HDL, and TG using Hitachi-917 analyzers (GmbH Diagnostic, Mannheim, Germany). LDL-C levels were calculated using the Friedewald formula. Insulin and C-peptide concentrations were determined using enzyme-linked immunosorbent assay (ELISA) kits (Mercodia, Uppsala, Sweden).

Coagulation tests, including PT, aPTT, and international normalized ratio (INR), were performed using standard laboratory techniques. Additionally, cardiac markers—such as serum levels of myoglobin, NT-proBNP, and creatine kinase—were assessed using standard laboratory assays.

### SESN2 measurements in plasma

SESN2 levels in plasma were measured using ELISA kits (Bioassay Technology Laboratory, Shanghai, China), following the manufacturer’s instructions. Briefly, 40 µL of plasma was added to each microplate well and mixed with 10 µL of human SESN2 antibody. Standard concentrations of SESN2 were prepared to generate a standard curve. Next, 50 µL of the standards were added to the corresponding microplate wells.

Afterward, 50 µL of streptavidin-HRP was added to both the sample and standard wells, and the plate was incubated at 37 ^∘^C for 60 min. Following incubation, the wells were washed and incubated for an additional 10 min at 37 ^∘^C in the dark after adding 50 µL each of substrate solution A and substrate solution B. To stop the reaction, 50 µL of stop solution was added to each well. The plate was then read using a microplate reader at 450 nm within 10 min of adding the stop solution to determine the optical density (OD) value for each well.

### Ethical statement

The Institutional Review Boards (IRB) of QBB (#Ex-2021-QF-QBB-RES-ACC-00049-0173) and Qatar University (#QU-IRB 1624-E/21) granted regulatory approval for the study protocol. Furthermore, the study was approved by the Qatar University Institutional Biohazard Committee (IBC) under reference number QU-IBC-2021/046. Informed consent was obtained from all participants prior to data collection. To ensure confidentiality, all data provided by QBB was already anonymized in order to safeguard the confidentiality of the participants.

### Statistical analysis

All analyses were conducted using RStudio (version 2024.04, Posit PBC, Boston, MA, USA). A *P* value of ≤0.05 was considered statistically significant. Descriptive statistics, including means and standard deviations for continuous variables and frequencies with percentages for categorical variables, were used to characterize the study population.

For normally distributed data, a one-way ANOVA was used to evaluate differences in cardiovascular risk markers across SESN2 tertiles. For non-normally distributed data, the Kruskal–Wallis test was applied. Partial correlation analysis was performed to assess relationships between SESN2 concentrations and CVD risk factors. The association between SESN2 tertiles (independent variable) and cardiovascular risk markers (dependent variables) was analyzed using binomial logistic regression, adjusted for age, gender, and nationality. SESN2 tertiles were defined as follows: tertile 1 (T1), tertile 2 (T2), and tertile 3 (T3), with T1 serving as the reference group.

All CVD risk factors were recoded into dichotomous variables, with high-risk cutoff values defined according to the literature: BMI (>30 kg/m^2^) [14], fasting glucose (>5.6 mmol/L) [15], insulin (>12 mIU/L), HbA1c (>6.5%) [16], HOMA-IR (>2), C-Peptide (>2.0 ng/mL), total cholesterol (>6.21 mmol/L), HDL cholesterol (<1 mmol/L), LDL cholesterol (>3 mmol/L), TG (>1.7 mmol/L), TG/HDL ratio (>2), TyG index (>8), SBP (>120 mm Hg), DBP (>80 mm Hg), PR (>80 bpm), PPI (>0.6), PWV (>9.3 m/s), NT-proBNP (>125 pg/mL), PT (>13.5 s), international normalized ratio (INR) (>1.1), aPTT (>35 s), fibrinogen (>4 g/L), PI (>1.5), mean IMT (>0.6 mm), and 10-year atherosclerotic cardiovascular disease (ASCVD) risk score (>7.5).

## Results

### Demographic and baseline characteristics of study participants

The baseline characteristics of the 844 study participants, including the healthy cohort (*N* ═ 326) and the diabetic cohort (*N* ═ 518), are summarized in Table 1. The healthy participants had a mean age of 35.37 ± 11.85 years, with 53% being female and 47% male. Notably, 59% of the healthy participants were from Qatar. The mean BMI for this group was 28.63 ± 5.74 kg/m^2^. In comparison, the diabetic participants were older, with a mean age of 49.87 ± 11.20 years. Males made up the majority of this group (57%). As expected, the diabetic group exhibited a significantly higher BMI than the healthy group (30.76 ± 5.74 kg/m^2^, *P* < 0.001). They also had elevated glucose levels (>5.5 mmol/L), with an average glucose value of 9.80 ± 3.93 mmol/L. Furthermore, diabetic individuals displayed higher HbA1c levels (>5.7%), while healthy participants maintained levels within the normal range, with an average HbA1c of 5.33 ± 0.38%. Diabetic participants also had markedly higher mean insulin levels of 23.54 ± 52.21 µU/mL, exceeding the normal range of 3.3–5.5 µU/mL.

**Table 1:** Baseline characteristics and demographics of the study population

**Characteristic**	**Overall *N* ═ 844^1^**	**Diabetic patients *N* ═ 518^1^**	**Healthy subjects *N* ═ 326^1^**	***P* value^2^**
Sestrin2 levels	6.55 (6.75)	5.49 (5.94)	8.25 (7.57)	0.001
*Demographics*				
Gender				0.004
Female	393 (47%)	221 (43%)	172 (53%)	
Male	451 (53%)	297 (57%)	154 (47%)	
Nationality				0.001
Non-Qatari	237 (28%)	102 (20%)	135 (41%)	
Qatari	607 (72%)	416 (80%)	191 (59%)	
Age	44.27 (13.46)	49.87 (11.20)	35.37 (11.85)	0.001
*Metabolic markers*				
BMI	29.94 (5.72)	30.76 (5.56)	28.63 (5.74)	0.001
Glucose	7.89 (3.93)	9.80 (3.93)	4.87 (0.67)	0.001
HbA1C	7.22 (2.02)	8.41 (1.70)	5.33 (0.38)	0.001
Insulin	19.89 (42.17)	23.54 (52.21)	14.17 (15.53)	0.001
HOMA-IR	7.44 (16.31)	10.07 (20.01)	3.31 (5.28)	0.001
C-Peptide	2.54 (1.42)	2.63 (1.40)	2.39 (1.44)	0.001
*Cardiovascular markers*				
Cholesterol-total	4.78 (1.00)	4.79 (1.08)	4.78 (0.87)	0.7
HDL cholesterol	1.30 (0.36)	1.25 (0.35)	1.38 (0.36)	0.001
LDL cholesterol	2.81 (0.89)	2.77 (0.96)	2.88 (0.76)	0.018
Triglycerides	1.52 (0.99)	1.75 (1.06)	1.15 (0.73)	0.001
TG/HDL ratio	1.34 (1.15)	1.58 (1.21)	0.95 (0.92)	0.001
TyG index	8.91 (0.81)	9.31 (0.70)	8.28 (0.51)	0.001
Systolic blood pressure	119.32 (14.85)	123.50 (14.63)	112.69 (12.62)	0.001
Diastolic blood pressure	69.58 (10.18)	71.01 (10.15)	67.31 (9.84)	0.001
Pulse rate	74.80 (11.00)	76.14 (10.78)	72.69 (11.03)	0.001
Pulse pressure index	1.23 (0.21)	1.25 (0.16)	1.21 (0.27)	0.001
Pulse wave velocity	13.98 (8.31)	15.48 (7.56)	11.60 (8.89)	0.001
NT-proBNP	42.87 (152.98)	50.65 (196.04)	31.18 (25.24)	0.7
10-year ASCVD risk	5.90 (8.67)	9.46 (9.92)	1.11 (2.02)	0.001
Lifetime ASCVD risk	40.65 (18.49)	51.44 (11.55)	26.15 (15.98)	0.001
*Coagulation tests*				
Prothrombin time (PT)	11.81 (2.65)	11.55 (3.27)	12.23 (0.94)	0.001
International normalization ratio (INR)	1.02 (0.22)	1.00 (0.28)	1.06 (0.08)	0.001
Activated partial thromboplastin time	32.82 (3.23)	32.78 (3.39)	32.87 (2.95)	0.4
Fibrinogen	3.38 (0.62)	3.46 (0.61)	3.26 (0.63)	0.001
*Carotid Doppler (common carotid arteries)*				
Pulsatility index (left)	2.04 (0.71)	2.10 (0.74)	1.71 (0.43)	0.009
Pulsatility index (right)	2.01 (0.61)	2.08 (0.62)	1.61 (0.35)	0.001
Mean intima-media thickness (left)	0.60 (0.14)	0.65 (0.14)	0.52 (0.09)	0.001
Mean intima-media thickness (right)	0.57 (0.13)	0.61 (0.14)	0.51 (0.10)	0.001

^1^*n* (%); Mean (SD); ^2^Pearson’s chi-squared test; Kruskal–Wallis rank sum test. BMI: Body mass index; HbA1C: Hemoglobin A1c; HDL: High-density lipoprotein cholesterol; LDL: Low-density lipoprotein cholesterol; TG/HDL: Triglycerides-to-HDL ratio; TyG: Triglyceride-glucose index; NT-proBNP: N-terminal-prohormone B-type natriuretic peptide; ASCVD: Atherosclerotic cardiovascular disease.

Regarding SESN2 levels, the diabetic group exhibited significantly lower plasma SESN2 levels compared to the healthy group (5.49 ± 5.94 vs 8.25 ± 7.57 ng/mL, *p* < 0.001) (Table 1). Cardiovascular parameters revealed differences between the two groups. Healthy participants had a mean SBP of 112.69 ± 12.62 mm Hg, DBP of 67.31 ± 9.84 mm Hg, and a PR of 72.69 ± 11.03 bpm. Conversely, the diabetic group had elevated SBP (123.50 ± 14.63 mm Hg), DBP (71.01 ± 10.15 mm Hg), and PR (76.14 ± 10.78 bpm). Additionally, the diabetic cohort had significantly higher 10-year and lifetime ASCVD (atherosclerotic cardiovascular disease) risks compared to the healthy cohort (9.46 ± 9.92 vs 1.11 ± 2.02, *P* < 0.001 and 51.44 ± 11.55 vs 26.15 ± 15.98, *P* < 0.001, respectively). Both cohorts showed coagulation parameters within normal ranges. The mean PT was 11.81 ± 2.65 s (typical range: 11–13 s), and the average INR was 1.02 ± 0.22 (normal range of values below 1.1).

Carotid Doppler analysis indicated that the diabetic group had significantly thicker left common carotid artery walls compared to the healthy group (0.65 ± 0.14 vs 0.52 ± 0.09, *P* < 0.001). Similar findings were observed for the right common carotid artery, where IMT values were higher in diabetics than in healthy participants (0.61 ± 0.14 vs 0.51 ± 0.10, *P* < 0.001)

### Comparison of cardiovascular risk markers across SESN2 tertiles in healthy and diabetic participants

The distribution of CVD risk indicators in healthy controls and diabetic subjects, stratified by SESN2 tertiles, is presented in Tables 2 and 3. Study participants were divided into three groups based on their SESN2 levels: Tertile 1 (T1, lowest), Tertile 2 (T2, intermediate), and Tertile 3 (T3, highest).

**Table 2:** Distribution of CVD risk markers among Sestrin2 tertiles in healthy controls

**Characteristic**	**Sestrin2 tertiles**			***P* value^2^**
	**T1** ***N* ═ 97^1^**	**T2** ***N* ═ 99^1^**	**T3** ***N* ═ 134^1^**	
Sestrin2 levels	2.49 (1.29)	5.26 (1.02)	16.98 (7.11)	0.001
*Demographics*				
Gender				0.4
Female	54 (50%)	55 (51%)	63 (58%)	
Male	55 (50%)	53 (49%)	46 (42%)	
Nationality				0.4
Non-Qatari	51 (47%)	41 (38%)	43 (39%)	
Qatari	58 (53%)	67 (62%)	66 (61%)	
Age	36.14 (12.73)	34.82 (12.69)	35.14 (10.01)	0.6
*Metabolic markers*				
BMI	29.25 (6.13)	28.06 (5.66)	28.57 (5.42)	0.5
Glucose	4.85 (0.62)	4.82 (0.55)	4.92 (0.80)	0.9
HbA1C	5.43 (0.38)	5.29 (0.40)	5.28 (0.35)	0.007
Insulin	16.36 (13.65)	13.18 (11.07)	12.95 (20.25)	0.005
HOMA-IR	3.75 (3.82)	2.92 (2.80)	3.25 (7.80)	0.017
C-peptide	2.66 (1.49)	2.30 (1.25)	2.21 (1.55)	0.014
*Cardiovascular markers*				
Total cholesterol	4.71 (0.86)	4.78 (0.88)	4.85 (0.87)	0.5
HDL cholesterol	1.35 (0.34)	1.39 (0.38)	1.41 (0.37)	0.5
LDL cholesterol	2.80 (0.78)	2.87 (0.74)	2.97 (0.76)	0.3
Triglycerides	1.24 (0.77)	1.18 (0.90)	1.04 (0.44)	0.4
TG/HDL ratio	1.06 (0.83)	0.99 (1.26)	0.82 (0.50)	0.3
TyG index	8.33 (0.58)	8.28 (0.52)	8.23 (0.43)	0.6
Systolic blood pressure	114.50 (14.06)	111.91 (12.63)	111.65 (10.89)	0.3
Diastolic blood pressure	67.98 (10.85)	65.84 (9.31)	68.10 (9.18)	0.2
Pulse rate	73.09 (10.27)	72.26 (11.56)	72.72 (11.31)	0.7
Pulse pressure index	1.22 (0.37)	1.19 (0.16)	1.22 (0.24)	0.9
Pulse wave velocity	12.74 (14.73)	10.84 (2.81)	11.21 (3.27)	0.7
NT-proBNP	33.40 (26.01)	29.59 (24.05)	30.50 (25.72)	0.5
10-year ASCVD risk	1.32 (2.41)	1.19 (2.15)	0.82 (1.33)	0.5
Lifetime ASCVD risk	25.95 (16.57)	25.27 (15.89)	27.22 (15.56)	0.5
*Coagulation tests*				
Prothrombin time (PT)	12.30 (0.78)	12.24 (1.05)	12.15 (0.97)	0.9
International normalization ratio (INR)	1.06 (0.07)	1.05 (0.09)	1.05 (0.08)	0.7
Activated partial thromboplastin time (aPTT)	32.98 (2.95)	32.70 (3.41)	32.94 (2.43)	0.2
Fibrinogen	3.27 (0.66)	3.19 (0.63)	3.31 (0.59)	0.4
*Carotid Doppler (common carotid arteries)*				
Pulsatility index (left carotid)	1.72 (0.57)	1.54 (0.33)	1.76 (0.31)	0.6
Pulsatility index (right carotid)	1.67 (0.33)	1.40 (0.47)	1.63 (0.33)	0.5
Mean intima-media thickness (left)	0.53 (0.10)	0.52 (0.10)	0.52 (0.08)	0.8
Mean intima-media thickness (right)	0.52 (0.10)	0.51 (0.09)	0.50 (0.10)	0.3

^1^*n* (%); Mean (SD); ^2^Pearson’s chi-squared test; Kruskal–Wallis rank sum test. CVD: Cardiovascular disease; BMI: Body mass index; HbA1C: Hemoglobin A1c; HDL: High-density lipoprotein cholesterol; LDL: Low-density lipoprotein cholesterol; TG/HDL: Triglycerides-to-HDL ratio; TyG: Triglyceride-glucose index; NT-proBNP: N-terminal-prohormone B-type natriuretic peptide; ASCVD: Atherosclerotic cardiovascular disease.

**Table 3:** Distribution of CVD risk markers among Sestrin2 tertiles in diabetic patients

**Characteristic**	**Sestrin2 tertiles**			***P* value^2^**
	**T1** ***N* ═ 184^1^**	**T2** ***N* ═ 182^1^**	**T3** ***N* ═ 147^1^**	
Sestrin2 levels	1.59 (0.57)	3.32 (0.58)	11.54 (6.98)	0.001
*Demographics*				
Gender				0.3
Female	79 (46%)	65 (38%)	77 (45%)	
Male	94 (54%)	107 (62%)	96 (55%)	
Nationality				0.4
Non-Qatari	31 (18%)	40 (23%)	31 (18%)	
Qatari	142 (82%)	132 (77%)	142 (82%)	
Age	49.36 (12.05)	50.27 (10.50)	49.98 (11.04)	0.9
*Metabolic markers*				
BMI	29.94 (5.34)	31.21 (5.81)	31.13 (5.47)	0.082
Glucose	9.83 (3.92)	9.71 (4.01)	9.86 (3.88)	0.8
HbA1C	8.45 (1.66)	8.42 (1.74)	8.37 (1.72)	0.7
Insulin	25.06 (65.82)	22.77 (41.30)	22.79 (46.65)	0.008
HOMA-IR	9.94 (20.45)	9.98 (17.84)	10.27 (21.62)	0.037
C-peptide	2.41 (1.16)	2.77 (1.60)	2.72 (1.37)	0.052
*Cardiovascular markers*				
Total cholesterol	4.87 (1.15)	4.73 (1.12)	4.76 (0.96)	0.6
HDL cholesterol	1.29 (0.37)	1.22 (0.30)	1.24 (0.36)	0.2
LDL cholesterol	2.87 (1.02)	2.72 (0.97)	2.71 (0.88)	0.3
Triglycerides	1.65 (0.95)	1.78 (1.20)	1.80 (1.03)	0.2
TG/HDL ratio	1.48 (1.13)	1.60 (1.24)	1.67 (1.25)	0.14
TyG index	9.26 (0.70)	9.31 (0.68)	9.36 (0.71)	0.3
Systolic blood pressure	121.47 (13.27)	126.24 (15.74)	122.79 (14.46)	0.017
Diastolic blood pressure	70.64 (9.50)	72.38 (10.66)	70.01 (10.15)	0.088
Pulse rate	76.66 (10.91)	75.31 (10.93)	76.44 (10.49)	0.4
Pulse pressure index	1.25 (0.19)	1.25 (0.15)	1.25 (0.14)	0.9
Pulse wave velocity	16.13 (11.14)	15.58 (5.56)	14.74 (4.05)	0.3
NT-proBNP	43.68 (118.88)	77.18 (317.26)	31.76 (33.09)	0.2
10-year ASCVD risk	8.94 (9.82)	10.01 (10.08)	9.43 (9.89)	0.4
Lifetime ASCVD risk	50.61 (11.41)	53.31 (11.94)	50.43 (11.14)	0.069
*Coagulation tests*				
Prothrombin time (PT)	11.59 (0.98)	11.67 (5.55)	11.40 (0.88)	0.002
International normalization ratio (INR)	1.01 (0.09)	1.01 (0.47)	0.99 (0.08)	0.010
Activated partial thromboplastin time (aPTT)	33.03 (2.86)	32.87 (3.99)	32.45 (3.23)	0.14
Fibrinogen	3.39 (0.54)	3.51 (0.66)	3.48 (0.62)	0.3
*Carotid Doppler (common carotid arteries)*				
Pulsatility index (left carotid)	2.16 (0.95)	2.15 (0.65)	1.98 (0.48)	0.7
Pulsatility index (right carotid)	2.07 (0.65)	2.07 (0.58)	2.10 (0.64)	>0.9
Mean intima-media thickness (left)	0.66 (0.14)	0.66 (0.15)	0.63 (0.13)	0.2
Mean intima-media thickness (right)	0.60 (0.14)	0.63 (0.14)	0.60 (0.13)	0.2

^1^*n* (%); mean (SD); ^2^Pearson’s chi-squared test; Kruskal–Wallis rank sum test. CVD: Cardiovascular disease; BMI: Body mass index; HbA1C: Hemoglobin A1c; HDL: High-density lipoprotein cholesterol; LDL: Low-density lipoprotein cholesterol; TG/HDL: Triglycerides-to-HDL ratio; TyG: Triglyceride-glucose index; NT-proBNP: N-terminal-prohormone B-type natriuretic peptide; ASCVD: Atherosclerotic cardiovascular disease.

In healthy controls, SESN2 levels varied significantly across tertiles, with T1 showing the lowest levels (2.49 ± 1.29) and T3 showing the highest (16.98 ± 7.11) (*P* < 0.001) (Table 2). Similarly, SESN2 levels in the diabetic cohort also differed significantly across tertiles (*P* < 0.001) (Table 3).

For healthy controls, BMI and glucose levels did not differ significantly among tertiles. However, significant differences were observed in HbA1c (*P* ═ 0.007), insulin (*P* ═ 0.005), HOMA-IR (*P* ═ 0.017), and c-peptide levels (*P* ═ 0.014), with the highest values observed in T1 compared to T2 and T3. Cardiovascular markers, including total cholesterol, HDL, LDL, TG, TG/HDL ratio, and the TyG index, showed no significant differences across tertiles. Although NT-proBNP levels were higher in T1, the difference was not statistically significant. Additionally, coagulation tests and Doppler studies of the common carotid arteries revealed no significant differences among tertiles (Table 2).

In contrast, diabetic participants exhibited a near-significant difference in BMI across tertiles (*P* ═ 0.082). Significant differences were noted for insulin (*P* ═ 0.008), HOMA-IR (*P* ═ 0.037), and c-peptide levels (*P* ═ 0.052). Furthermore, SBP (*P* ═ 0.017), diastolic blood pressure (*P* ═ 0.088), lifetime ASCVD risk (*P* ═ 0.069), PT (*P* ═ 0.002), and international normalized ratio (INR) (*P* ═ 0.010) differed significantly among SESN2 tertiles. As in the healthy group, Doppler studies of the common carotid arteries did not show statistically significant differences among tertiles (Table 3).

### Based on binomial correlation between CVD risk markers and SESN2 levels in healthy and diabetic study participants

The correlations between CVD risk indicators and SESN2 levels in healthy subjects are summarized in Table 4. A significant inverse relationship was identified between SESN2 levels and several markers, including HbA1c (−0.19, *P* ═ 0.0006), insulin (−0.19, *P* ═ 0.0006), HOMA-IR (−0.17, *P* ═ 0.0024), and C-peptide (−0.18, *P* ═ 0.0012). A modest negative correlation was also noted between SESN2 levels and TG (−0.10, *P* ═ 0.0614), while a statistically significant negative correlation was observed with the TG/HDL ratio (−0.12, *P* ═ 0.0283). No significant associations were found between SESN2 levels and the results of coagulation tests within the healthy cohort.

**Table 4:** Partial correlation between Sestrin2 levels and CVD risk markers among healthy controls

**Characteristic**	**Correlation coefficient**	***P* value**	**R^2^**
*Metabolic markers*			
BMI	−0.09	0.1050	0.0082
Glucose	−0.01	0.9104	0.0000
HbA1C	−0.19	0.0006	0.0365
Insulin	−0.19	0.0006	0.0359
HOMA-IR	−0.17	0.0024	0.0284
C-peptide	−0.18	0.0012	0.0322
*Cardiovascular markers*			
Total cholesterol	0.09	0.1065	0.0081
HDL cholesterol	0.09	0.0910	0.0089
LDL cholesterol	0.10	0.0778	0.0097
Triglycerides	−0.10	0.0614	0.0109
TG/HDL ratio	−0.12	0.0283	0.0149
TyG index	−0.09	0.1257	0.0073
Systolic blood pressure	−0.08	0.1658	0.0060
Diastolic blood pressure	0.04	0.5232	0.0013
Pulse rate	−0.05	0.3394	0.0028
Pulse pressure index	0.01	0.8913	0.0001
Pulse wave velocity	0.03	0.6399	0.0007
NT-proBNP	−0.01	0.8398	0.0001
10-year ASCVD risk	−0.05	0.3740	0.0025
Lifetime ASCVD risk	0.06	0.2558	0.0040
*Coagulation tests*			
Prothrombin time (PT)	−0.03	0.5556	0.0011
International normalization ratio (INR)	0.00	0.9726	0.0000
Activated partial thromboplastin time (aPTT)	0.02	0.6843	0.0005
Fibrinogen	−0.03	0.5620	0.0010
*Carotid Doppler (common carotid arteries)*			
Pulsatility index (left carotid)	−0.15	0.0063	0.0230
Pulsatility index (right carotid)	−0.15	0.0060	0.0233
Mean intima-media thickness (left carotid)	0.04	0.5251	0.0013
Mean intima-media thickness (right carotid)	0.01	0.7954	0.0002

CVD: Cardiovascular disease; BMI: Body mass index; HbA1C: Hemoglobin A1c; HDL: High-density lipoprotein cholesterol; LDL: Low-density lipoprotein cholesterol; TG/HDL: Triglycerides-to-HDL ratio; TyG: Triglyceride-glucose index; NT-proBNP: N-terminal-prohormone B-type natriuretic peptide; ASCVD: Atherosclerotic cardiovascular disease.

Doppler studies of the common carotid arteries revealed a significant negative correlation between SESN2 levels and the PI of both the left (−0.15, *P* ═ 0.0063) and right (−0.15, *P* ═ 0.0060) carotid arteries.

Table 5 outlines the correlations between CVD risk markers and SESN2 levels in individuals with diabetes. Unlike the healthy group, BMI demonstrated a positive correlation with SESN2 levels in diabetic participants. Although glucose (−0.02, *P* ═ 0.6248) and HbA1c (−0.04, *P* ═ 0.3091) were not significantly correlated with SESN2, significant positive correlations were observed for insulin (0.15, *P* ═ 0.0005), HOMA-IR (0.11, *P* ═ 0.0106), and C-peptide (0.12, *P* ═ 0.0048).

**Table 5:** Partial correlation between Sestrin2 levels and CVD risk markers among diabetic patients

**Characteristic**	**Correlation coefficient**	***P* value**	**R^2^**
*Metabolic markers*			
BMI	0.08	0.0573	0.0070
Glucose	−0.02	0.6248	0.0005
HbA1C	−0.04	0.3091	0.0020
Insulin	0.15	0.0005	0.0235
HOMA-IR	0.11	0.0106	0.0127
C-peptide	0.12	0.0048	0.0154
*Cardiovascular markers*			
Total cholesterol	−0.01	0.7791	0.0002
HDL cholesterol	−0.07	0.0901	0.0056
LDL cholesterol	−0.05	0.2924	0.0022
Triglycerides	0.08	0.0619	0.0068
TG/HDL ratio	0.09	0.0445	0.0078
TyG index	0.06	0.2065	0.0031
Systolic blood pressure	0.04	0.3124	0.0020
Diastolic blood pressure	0.00	0.9868	0.0000
Pulse rate	0.01	0.8054	0.0001
Pulse pressure index	−0.02	0.6871	0.0003
Pulse wave velocity	0.02	0.6929	0.0003
NT-proBNP	−0.04	0.3213	0.0019
10-year ASCVD risk	0.02	0.6538	0.0004
Lifetime ASCVD risk	−0.01	0.8484	0.0001
*Coagulation tests*			
Prothrombin time (PT)	−0.07	0.0928	0.0055
International normalization ratio (INR)	−0.07	0.1220	0.0047
Activated partial thromboplastin time (aPTT)	−0.07	0.1036	0.0052
Fibrinogen	0.04	0.3247	0.0019
*Carotid Doppler (common carotid arteries)*			
Pulsatility index (left carotid)	0.00	0.9450	0.0000
Pulsatility index (right carotid)	0.00	0.9524	0.0000
Mean intima-media thickness (left carotid)	−0.01	0.8993	0.0000
Mean intima-media thickness (right carotid)	0.03	0.4860	0.0009

CVD: Cardiovascular disease; BMI: Body mass index; HbA1C: Hemoglobin A1c; HDL: High-density lipoprotein cholesterol; LDL: Low-density lipoprotein cholesterol; TG/HDL: Triglycerides-to-HDL ratio; TyG: Triglyceride-glucose index; NT-proBNP: N-terminal-prohormone B-type natriuretic peptide; ASCVD: Atherosclerotic cardiovascular disease.

Furthermore, while there was a trend toward a negative correlation between SESN2 levels and HDL cholesterol (−0.07, *P* ═ 0.0901), significant positive correlations were observed for TG (0.08, *P* ═ 0.0619) and the TG/HDL ratio (0.09, *P* ═ 0.0445). Other indicators, including total cholesterol (−0.01, *P* ═ 0.7791), LDL cholesterol (−0.05, *P* ═ 0.2924), NT-proBNP (−0.04, *P* ═ 0.3213), lifetime CVD risk (−0.01, *P* ═ 0.8484), PT (−0.07, *P* ═ 0.0.0928), and INR (−0.07, *P* ═ 0.1220), showed no statistically significant correlations. Similarly, no significant associations were observed between SESN2 levels and the IMT of the carotid arteries.

### Association between CVD risk markers and SESN2 tertiles in healthy and diabetic study participants

Based on binomial logistic regression adjusted for age, gender, and nationality, Table 6 presents the relationships between CVD risk markers and SESN2 tertiles in healthy individuals. Subjects in the highest tertile (T3) had significantly lower odds of elevated HbA1C (adjusted odds ratio [AOR] ═ 0.33, *P* ═ 0.00), insulin (AOR ═ 0.23, *P* ═ 0.00), HOMA-IR (AOR ═ 0.58, *P* ═ 0.06), and C-peptide (AOR ═ 0.56, *P* ═ 0.04). Likewise, T3 membership reduced the odds of high TG (AOR ═ 0.37, *P* ═ 0.03). However, no significant associations were observed between SESN2 tertiles and other CVD markers, including coagulation tests or Doppler measures of the common carotid arteries.

**Table 6:** Association between Sestrin2 tertiles and CVD risk markers among healthy controls using T1 as reference category (adjusted for gender, nationality, and age)

**Variable**	**T2**			**T3**		
	**AOR**	**95% CI**	***P* value**	**AOR**	**95% CI**	***P* value**
*Metabolic markers*						
High BMI	0.77	0.40–1.47	0.42	0.82	0.43–1.58	0.56
High glucose	1.34	0.56–3.22	0.51	1.66	0.70–3.94	0.25
High HbA1C	0.30	0.14–0.63	0.00	0.33	0.16–0.69	0.00
High insulin	0.59	0.27–1.28	0.18	0.23	0.08–0.63	0.00
High HOMA-IR	0.62	0.35–1.10	0.10	0.58	0.33–1.01	0.06
High C-peptide	0.58	0.33–1.03	0.06	0.56	0.32–0.98	0.04
*Cardiovascular markers*						
High total cholesterol	1.09	0.58–2.02	0.79	1.45	0.79–2.65	0.23
Low HDL cholesterol	0.91	0.47–1.77	0.78	1.05	0.55–2.00	0.89
High LDL cholesterol	1.28	0.72–2.29	0.40	1.51	0.84–2.70	0.17
High triglycerides	0.82	0.38–1.77	0.62	0.37	0.15–0.92	0.03
High TG/HDL ratio	0.36	0.04–3.60	0.38	0.39	0.04–3.86	0.42
High TyG index	1.36	0.73–2.53	0.33	1.50	0.81–2.80	0.20
High systolic blood pressure	0.99	0.49–2.01	0.99	0.72	0.35–1.47	0.37
High diastolic blood pressure	0.44	0.16–1.18	0.10	1.33	0.57–3.08	0.51
High pulse rate	1.03	0.54–1.98	0.92	1.06	0.56–2.02	0.86
High pulse wave velocity	0.82	0.28–2.41	0.72	0.49	0.18–1.34	0.16
High NT-proBNP	0.89	0.05–14.86	0.93	0.91	0.05–15.26	0.95
*Coagulation tests*						
High prothrombin time (PT)	1.26	0.44–3.58	0.66	0.99	0.33–2.99	0.98
High international normalization ratio (INR)	0.83	0.47–1.45	0.52	1.19	0.68–2.07	0.55
High activated partial thromboplastin time (aPTT)	0.86	0.42–1.76	0.68	1.06	0.53–2.12	0.87
High fibrinogen	0.66	0.29–1.50	0.32	0.72	0.33–1.59	0.42
*Carotid Doppler (common carotid arteries)*						
High pulsatility index (left carotid)	0.39	0.01–11.25	0.58	0.76	0.04–15.06	0.85
High pulsatility index (right carotid)	0.83	0.06–12.18	0.89	0.91	0.10–8.36	0.94
High mean intima-media thickness (left)	1.47	0.61–3.54	0.39	1.30	0.53–3.21	0.56
High mean intima-media thickness (right)	0.93	0.35–2.51	0.89	1.16	0.44–3.07	0.76

CI: Confidence interval; AOR: Adjusted odds ratio; CVD: Cardiovascular disease; BMI: Body mass index; HbA1C: Hemoglobin A1c; HDL: High-density lipoprotein cholesterol; LDL: Low-density lipoprotein cholesterol; TG/HDL: Triglycerides-to-HDL ratio; TyG: Triglyceride-glucose index; NT-proBNP: N-terminal-prohormone B-type natriuretic peptide.

Table 7 focuses on participants with diabetes and highlights different trends. Here, being in T3 was associated with significantly higher odds of having a high BMI (AOR ═ 1.96, *P* ═ 0.05), high TG (AOR ═ 1.57, *P* ═ 0.04), high NT-proBNP (AOR ═ 7.27, *P* ═ 0.01), and high fibrinogen (AOR ═ 1.92, *P* ═ 0.03). Notably, subjects in T2 also had increased odds of high fibrinogen (AOR ═ 2.26, *P* ═ 0.01) and high SBP (AOR ═ 1.75, *P* ═ 0.02). Conversely, T3 membership significantly reduced the likelihood of elevated INR (AOR ═ 0.64, *P* ═ 0.09) and increased IMT of the left carotid artery (AOR ═ 0.63, *P* ═ 0.09). Additionally, T2 was associated with a marked reduction in high INR (AOR ═ 0.43, *P* ═ 0.00).

No significant associations were observed between SESN2 tertiles and total cholesterol, HDL, LDL, TyG index, DBP, PR, pulse wave velocity, PT, aPTT, PI, or right carotid IMT.

**Table 7:** Association between Sestrin2 tertiles and CVD risk markers among diabetic patients using T1 as reference category (adjusted for gender, nationality, and age)

**Variable**	**T2**			**T3**		
	**AOR**	**95% CI**	***P* value**	**AOR**	**95% CI**	***P* value**
*Metabolic markers*						
High BMI	1.86	0.96–3.62	0.07	1.96	1.00–3.84	0.05
High glucose	0.71	0.30–1.65	0.42	0.90	0.37–2.19	0.82
High HbA1C	1.00	0.68–1.68	1.00	1.00	0.54–1.32	1.00
High insulin	0.96	0.57–1.64	0.89	1.13	0.67–1.88	0.65
High HOMA-IR	0.83	0.42–1.63	0.59	1.76	0.80–3.85	0.16
High C-peptide	1.21	0.77–1.90	0.41	1.40	0.89–2.22	0.15
*Cardiovascular markers*						
High total cholesterol	0.87	0.56–1.37	0.55	0.96	0.62–1.50	0.86
Low HDL cholesterol	1.05	0.67–1.64	0.84	0.93	0.59–1.46	0.75
High LDL cholesterol	0.70	0.45–1.09	0.11	0.72	0.46–1.11	0.13
High triglycerides	1.23	0.79–1.91	0.36	1.57	1.02–2.43	0.04
High TG/HDL ratio	1.12	0.53–2.34	0.77	1.22	0.59–2.53	0.59
High TyG index	1.26	0.20–7.85	0.80	0.68	0.15–3.14	0.62
High systolic blood pressure	1.75	1.11–2.75	0.02	1.21	0.78–1.90	0.40
High diastolic blood pressure	1.28	0.77–2.15	0.34	0.91	0.53–1.57	0.74
High pulse rate	0.88	0.56–1.40	0.60	0.94	0.60–1.47	0.77
High pulse wave velocity	0.39	0.08–1.85	0.23	2.20	0.21–22.58	0.51
High NT-proBNP	1.80	0.67–4.85	0.24	0.42	0.05–1.21	0.08
*Coagulation tests*						
High prothrombin time (PT)	0.43	0.11–1.71	0.23	0.42	0.11–1.64	0.21
High international normalization ratio (INR)	0.43	0.24–0.75	0.00	0.64	0.38–1.07	0.09
High activated partial thromboplastin time (aPTT)	0.98	0.58–1.64	0.94	0.90	0.53–1.51	0.69
High fibrinogen	2.26	1.27–4.01	0.01	1.92	1.08–3.41	0.03
*Carotid Doppler (common carotid arteries)*						
High pulsatility index (left carotid)	1.19	0.29–4.82	0.81	0.65	0.19–2.26	0.50
High pulsatility index (right carotid)	1.43	0.36–5.71	0.61	0.83	0.25–2.82	0.77
High mean intima-media thickness (left carotid)	1.13	0.65–1.96	0.66	0.63	0.37–1.08	0.09
High mean intima-media thickness (right carotid)	1.08	0.64–1.83	0.78	0.88	0.52–1.51	0.65

CI: Confidence interval; AOR: Adjusted odds ratio; CVD: Cardiovascular disease; BMI: Body mass index; HbA1C: Hemoglobin A1c; HDL: High-density lipoprotein cholesterol; LDL: Low-density lipoprotein cholesterol; TG/HDL: Triglycerides-to-HDL ratio; TyG: Triglyceride-glucose index; NT-proBNP: N-terminal-prohormone B-type natriuretic peptide.

## Discussion

The present study provides valuable insights into the association between circulating SESN2 levels and the risk of CVD in both healthy individuals and patients with T2DM. Our observations revealed a notable decrease in SESN2 levels among diabetic patients compared to healthy controls. Furthermore, the inverse relationship between SESN2 levels and several CVD risk factors—including HbA1c, insulin, HOMA-IR, C-peptide, TG, TG/HDL ratio, and pulsatility—suggests that decreased SESN2 levels could be a potential indicator of increased CVD risk in healthy individuals.

T2DM, a metabolic disorder characterized by hyperglycemia and insulin resistance, is a growing global epidemic, with its prevalence rising rapidly among adults. The primary cause of morbidity and mortality in diabetic individuals is cardiovascular events [17]. Excessive ROS production in diabetes contributes to CVD pathogenesis; mitigating ROS accumulation is therefore critical for preserving cardiovascular health [18]. SESN2, a stress-inducible antioxidant protein, plays a pivotal role in defending against oxidative stress caused by ROS accumulation and protecting against CVD [6]. Previous studies have demonstrated significantly elevated serum SESN2 levels in patients with coronary artery disease (CAD) compared to those without [19, 20], suggesting a protective role in these patients. These findings align with our current investigation of SESN2’s association with CVD risk factors.

One key finding of our study is the significantly lower SESN2 levels observed in the diabetic cohort compared to healthy controls. Experimentally, we have previously shown that SESN2 deletion exacerbates oxidative stress and endothelial cell death in response to ER stress, a mechanism triggered by diabetes that plays a critical role in cardiovascular dysfunction [21, 22]. These results are consistent with prior reports [23, 24]. In contrast, Chung et al. [25] observed higher SESN2 levels in diabetic patients compared to healthy controls. This discrepancy may be due to their study’s focus on newly diagnosed diabetic patients, where elevated SESN2 levels may represent a compensatory response in the early stages of the disease. However, as diabetes progresses, this compensatory mechanism could become overwhelmed, leading to reduced SESN2 levels as the disease becomes more established.

Our findings provide important insights into the complex relationship between plasma SESN2 levels and various CVD risk factors in healthy and diabetic adults from the Qatari population. In healthy individuals, our results show that HbA1c, insulin, HOMA-IR, and C-peptide levels were highest in the lowest SESN2 tertile (T1). Correlation analyses further supported these observations, with a significant negative correlation between SESN2 levels and HbA1c, insulin, HOMA-IR, and C-peptide. These findings are consistent with those of Mohany et al. [24], who reported negative correlations between SESN2 levels and glucose, HbA1c, total cholesterol, and LDL. Furthermore, healthy controls in the highest SESN2 tertile (T3) exhibited lower odds of elevated HbA1c, insulin, HOMA-IR, and C-peptide. These results suggest that SESN2 may play a role in promoting glucose homeostasis and insulin sensitivity. Another notable finding was the inverse correlation between SESN2 levels and CVD risk markers, such as TG, TG/HDL ratio, and vascular pulsatility, suggesting that higher circulating SESN2 levels in healthy individuals may have a protective role in mitigating CVD development and progression.

Interestingly, SESN2 presented differently in the diabetic cohort. Patients in the second tertile (T2) showed the highest values for BMI, insulin, HOMA-IR, C-peptide, and both systolic and diastolic blood pressure. A significant positive correlation was observed between SESN2 levels and BMI, insulin, HOMA-IR, C-peptide, TG, and TG/HDL ratio in diabetic patients. These findings suggest that, under diabetic conditions, elevated SESN2 levels may reflect ongoing stress and cardiometabolic disturbances rather than effective protection. Higher SESN2 levels in diabetic participants were associated with increased odds of elevated BMI, TG, SBP, NT-proBNP, and fibrinogen. On the other hand, higher SESN2 levels were associated with lower odds of elevated INR and IMT. These results align with findings from Tian et al. [26], who reported a positive correlation between low SESN2 levels and increased CAD risk in diabetic individuals.

The dichotomy between healthy individuals and diabetic patients highlights the complexity of metabolic disturbances in diabetes. Elevated SESN2 levels in diabetic patients may reflect a compensatory response to chronic stress and metabolic dysregulation rather than a sign of effective protection. Initially, SESN2 levels rise to counteract stress, but as stress becomes persistent and excessive, SESN2 may lose its efficacy in mitigating damage and restoring homeostasis. Moreover, chronic hyperglycemia results in dysregulated metabolic pathways which might hinder SESN2 from exerting its protective abilities and results in a state where elevated levels of SESN2 do not correspond with effective metabolic regulation. Furthermore, elevated SESN2 levels do not necessarily indicate activity, rather than an impaired function against stress despite its presence. As the disease progresses, the initially elevated SESN2 levels might render ineffective leading to reduced activity despite its elevated serum level.

As with every study, our study has some limitations. The study focused exclusively on the Qatari population using subjects from the QBB biorepository, which may limit the generalizability of our findings to broader populations. Additionally, the cross-sectional design of the study prevents us from drawing causal inferences between SESN2 levels and CVD risk factors. Potential confounders, such as dietary habits and physical activity were also not accounted for.

In summary, our findings contribute to the growing body of evidence supporting the cardioprotective role of SESN2, particularly in healthy individuals. In the diabetic cohort, SESN2 appears to play a context-dependent role, with its effects influenced by factors, such as disease progression, individual characteristics, and comorbidities. The observed correlations between SESN2 and various metabolic health indicators highlight its potential as both a predictive biomarker for identifying individuals at increased risk of CVD and a target for interventions aimed at preventing CVD development and progression. Future studies investigating the mechanistic links between SESN2 and glucose, lipid metabolism, and oxidative response may reveal important insights into the potential therapeutic targeting of SESN2 for the management of diabetes and its associated cardiovascular complications. Future research should focus on understanding the mechanisms underlying its protective effects and exploring practical interventions that can modify SESN2 levels to promote cardiovascular health. Establishing a clearer connection between SESN2 modulation and improved CVD outcomes is expected to be significantly impactful in clinical practice and subsequent overall patient management strategies. Understanding SESN2 in the context of diabetes and its cardiovascular sequelae may open novel avenues for timely interventions and targeted therapies leading to ameliorated outcomes for individuals at risk.

## Conclusion

In conclusion, our study is the first to highlight the role of SESN2 as a biomarker for CVD risk associated with T2DM in the Middle Eastern population. The implications of our findings suggest that SESN2 could serve as a biomarker for the timely detection and assessment of CVD risk in healthy individuals and patients with diabetes and as a potential therapeutic target. The significant differences in the plasma levels of SESN2 between the diabetic and healthy groups, along with the trends observed in the correlation and association analyses with various CVD risk factors, emphasize the critical role that SESN2 plays in cardiovascular health, especially in patients with diabetes. This underscores the importance of establishing optimal cutoff values for SESN2 in both healthy and diabetic individuals to support the assessment of CVD risk, particularly in T2DM patients. Interventions aimed at maintaining sufficient SESN2 levels could provide a novel approach for mitigating cardiovascular risk in patients with diabetes. Future studies aimed at investigating the differences in SESN2 levels at different stages of diabetes may help to shed light on its potential and improve patient outcomes.

## Data Availability

All relevant data are included in the manuscript. Additional data and information can be provided by the corresponding author upon reasonable request.
